# MRI and CT features of head and neck myoepithelioma: comparison with parotid pleomorphic adenoma

**DOI:** 10.1007/s11604-025-01867-6

**Published:** 2025-09-15

**Authors:** Hiroki Kato, Takuya Seko, Hirofumi Shibata, Takenori Ogawa, Tomohiro Ando, Masaya Kawaguchi, Yoshifumi Noda, Abdelazim Elsayed Elhelaly, Hirohiko Imai, Masayuki Matsuo

**Affiliations:** 1 Department of Radiology, Gifu University, 1-1 Yanagido, Gifu, 501-1194 Japan; 2 Department of Otolaryngology, Gifu University, Gifu, Japan; 3 Department of Radiology, Massachusetts General Hospital, Harvard Medical School, 55 Fruit Street, White 270, Boston, MA 02114 USA; 4 Department of Frontier Science for Imaging, Gifu University, Gifu, Japan; 5 Department of Food Hygiene and Control, Faculty of Veterinary Medicine, Suez Canal University, Ismailia, Egypt; 6 Innovation Research Center for Quantum Medicine, Graduate School of Medicine, Gifu University, Gifu, Japan

**Keywords:** Myoepithelioma, Pleomorphic adenoma, Neck, MRI, CT

## Abstract

**Purpose:**

To evaluate the MRI and CT features of head and neck myoepithelioma in comparison with parotid pleomorphic adenoma.

**Methods:**

This retrospective study included 11 patients with histopathologically confirmed myoepithelioma of the head and neck and 103 patients with pleomorphic adenoma of the parotid gland, all of whom underwent preoperative MRI. Among them, seven patients with myoepithelioma and 29 with pleomorphic adenoma also underwent preoperative CT. MRI and CT findings were compared between the two groups.

**Results:**

Multinodular configuration (27% vs. 4%), mild hyperintensity relative to the spinal cord on T2-weighted images (91% vs. 48%), and focal unenhanced areas on fat-suppressed contrast-enhanced T1-weighted images (100% vs. 47%) were significantly more frequent in myoepithelioma than in pleomorphic adenoma, respectively (*p* < 0.05). In contrast, marked hyperintensity relative to the spinal cord on T2-weighted images (46% vs. 9%), higher signal intensity ratios on T2-weighted images (1.68 ± 0.47 vs. 1.40 ± 0.39), and higher apparent diffusion coefficient (ADC) values (1.68 ± 0.36 vs. 1.38 ± 0.23 × 10^−3^ mm^2^/s) were significantly more common in pleomorphic adenoma than in myoepithelioma, respectively (*p* < 0.05). Contrast-enhanced CT attenuation was significantly higher in myoepithelioma than in pleomorphic adenoma (93.3 ± 10.5 vs. 59.2 ± 22.8 HU, *p* < 0.05).

**Conclusions:**

Although MRI and CT features of myoepithelioma and pleomorphic adenoma can overlap, the presence of a multinodular configuration, focal unenhanced areas, lower T2 signal intensity, lower ADC values, and higher contrast-enhanced CT attenuation may aid in differentiating myoepithelioma from pleomorphic adenoma.

## Introduction

Myoepithelioma was formerly considered a variant of pleomorphic adenoma; however, it has been recognized as a distinct entity among salivary gland neoplasms by the World Health Organization classification in 1991. These are uncommon benign tumors, accounting for approximately 1.5% of all salivary gland tumors. They typically affect both major and minor salivary glands, with the parotid gland being the most common site (approximately 40% of cases), followed by the minor salivary glands. The incidence is similar in males and females, with cases reported across a wide age range (9–85 years), most commonly during the third decade of life. No definitive risk factors have been identified.

Histopathologically, myoepitheliomas consist almost entirely of myoepithelial cells. They are classified into spindle cell, epithelioid cell, clear cell, and mixed types. However, biopsy and fine-needle aspiration cytology can be challenging due to their nonspecific features and potential for misdiagnosis. Depending on the biopsy site, myoepitheliomas can histologically resemble other salivary gland tumors [1]. Therefore, a combination of architectural assessment and immunochemical profiling is crucial for accurate diagnosis [1].

Myoepitheliomas have a very low recurrence rate, particularly compared to pleomorphic adenomas, and recurrent cases reported to be associated with positive surgical margins. The recommended treatment is complete surgical excision with clear safety margins [2, 3]. In contrast, the recurrence rate for pleomorphic adenomas ranges from 20 to 45% following simple extracapsular dissection, 2 to 5% after lateral lobectomy of the parotid gland, and 0 to 0.4% after total parotidectomy [4]. Accordingly, superficial parotidectomy with facial nerve preservation is preferred for tumors in the superficial lobe, while total parotidectomy is required in cases involving the deep lobe [5].

Accurate preoperative differentiation between myoepithelioma and pleomorphic adenoma is essential due to differing recurrence risks and surgical approaches. Although several case reports [6–11] and case series [12, 13] have described imaging features of salivary gland myoepithelioma, to the best of our knowledge, no studies have yet comprehensively compared MRI and CT findings between myoepithelioma and pleomorphic adenoma. Hence, this study aims to evaluate the MRI and CT features of head and neck myoepithelioma in comparison with parotid pleomorphic adenoma.

## Methods

### Patients

The present study was approved by the human research committee of the institutional review board of our hospital and complied with the guidelines of the Health Insurance Portability and Accountability Act of 1996 and the Declaration of Helsinki. Due to the retrospective nature of the study, the requirement for informed consent was waived. We identified patients with histopathologically confirmed myoepithelioma of the head and neck and pleomorphic adenoma of the parotid gland from March 2009 to December 2024 using our hospital’s electronic medical records. Inclusion criteria were as follows: (a) preoperative MRI, (b) diagnosis based on surgical resection, and (c) primary tumor. Exclusion criteria included: (a) diagnosis based on biopsy and (b) recurrent tumors. A total of 11 patients with head and neck myoepithelioma and 103 patients with parotid pleomorphic adenoma who underwent preoperative MRI were included. Among the 11 patients, tumor locations in myoepithelioma included the parotid gland (*n* = 6), palate (*n* = 3), submandibular gland (*n* = 1), and oropharynx (*n* = 1).

### MRI imaging

MRI images were obtained from all 114 patients using a 1.5-T MRI system (Intera Achieva 1.5 T Pulsar or Ingenia Prodiva 1.5 T CS, Philips Healthcare, Best, The Netherlands) or a 3.0 Tesla MRI scanner (Intera Achieva 3.0 T Quasar Dual, Philips Healthcare, Best, The Netherlands). All images were obtained at a section thickness of 3–4 mm with an intersection gap of 1 mm. Axial non-fat-suppressed T2-weighted fast spin-echo (TR/TE, 3000–5710/90 ms), axial and coronal non-fat-suppressed T1-weighted spin-echo (TR/TE, 688–778/15–18 ms), and coronal fat-suppressed T2-weighted fast spin-echo (TR/TE, 3421–5329/60–80 ms) images were obtained from all patients. Axial short-tau inversion recovery single-shot spin-echo echo-planar diffusion-weighted images (TR/TE, 4000–6710/65–68 ms) with b value of 0 and 1000 s/mm were obtained from 103 patients (7 myoepitheliomas and 96 pleomorphic adenomas). Axial and coronal fat-suppressed contrast-enhanced T1-weighted spin-echo (TR/TE, 576–764/14–18 ms) images were obtained from 41 patients (5 myoepitheliomas and 36 pleomorphic adenomas) after the intravenous injection of 0.1 mmol/kg of gadopentetate dimeglumine (Magnevist; Bayer HealthCare, Leverkusen, Germany) or gadobutrol (Gadavist; Bayer HealthCare). Axial fat-suppressed dynamic contrast-enhanced T1-weighted gradient-echo (TR/TE, 3.7–5.9/1.8–3.9 ms) images were obtained from 21 patients (3 myoepitheliomas and 18 pleomorphic adenomas) before and 30, 60, 90, 120, 150, and 180 s after intravenous administration of contrast material at a rate of 2 ml/s. When the contrast enhancement was heterogeneous, the signal intensities (SIs) of solid components were measured, and time-intensity curves were constructed.

### CT imaging

CT imaging was performed for 36 patients (7 myoepitheliomas and 29 pleomorphic adenomas) using an 8-slice CT scanner (LightSpeed Ultra; GE Healthcare, Milwaukee, WI, USA), or 16-slice CT scanner (LightSpeed 16; GE Healthcare, Milwaukee, WI, USA), or 64-slice CT scanner (Brilliance 64; Philips Healthcare, Best, The Netherlands or Discovery CT750 HD; GE Healthcare, Milwaukee, WI, USA), or 256-slice CT scanner (Revolution CT with Apex Edition; GE Healthcare, Milwaukee, WI, USA). All transverse CT images were reconstructed with 2.5-mm section thickness and no overlap. Unenhanced CT images were obtained from all patients. Contrast-enhanced CT images were obtained for 13 patients (3 myoepitheliomas and 10 pleomorphic adenomas). Single-phase contrast-enhanced CT images were obtained 45 s after initiating an intravenous bolus injection of 100 mL of nonionic iodine contrast material containing 240 or 300 mg iodine/mL at an injection rate of 2 mL/s.

### Imaging assessment

Two radiologists (Radiologists 1 and 2) with 26 and 4 years of post-training experience in head and neck imaging, respectively, individually reviewed MRI and CT images while blinded to clinical and pathological data. The reviewers were unaware of any clinical information or pathological diagnosis. Disagreements between the two reviewers were resolved by consensus.

Qualitative MRI findings included anatomical location of the parotid gland (superficial/deep lobe and superior/inferior pole), lobulated margin, multinodular configuration, capsule formation (entire/partial/absence), intratumoral septa, heterogeneity on T1-, T2-, and fat-suppressed contrast-enhanced T1-weighted images, hyperintense areas on T1-weighted images, hypointense areas on T2-weighted images, predominant SI of solid components on T1-, T2-, and fat-suppressed contrast-enhanced T1-weighted images, and focal unenhanced areas. The parotid gland was anatomically divided into superficial and deep lobes based on the retromandibular vein. The superior and inferior poles were defined as the superior and inferior halves of the parotid gland, respectively. The presence of a capsule formation and intratumoral septa was assessed on T2-weighted images. The SI on T1-weighted images was categorized as hypo-, iso-, or hyperintense relative to the spinal cord; on T2-weighted images as hypo- to isointense, mild hyperintense, or markedly hyperintense relative to the spinal cord; and on fat-suppressed contrast-enhanced T1-weighted images as mild, moderate, or strong enhancement.

Time intensity curves were classified as the following three types:

- Type A: time of peak enhancement ≤ 120 s, washout ratio ≥ 30%
- Type B: time of peak enhancement ≤ 120 s, washout ratio < 30%
- Type C: time of peak enhancement > 120 s.

Washout ratios were calculated using the following formula:

$$\text{Washout ratio}= \frac{\text{maximum SI}-\text{SI at 3 min}}{\text{maximum SI}-\text{SI of precontrast images}} \times 100 \left({\%}\right).$$

Quantitative MRI measurements included maximum lesion diameter and SIs on axial T1-, T2-, and fat-suppressed contrast-enhanced T1-weighted images, with regions of interest (ROIs) placed on solid components of the lesions (avoiding fluid-filled or artifact-prone areas). ROIs were placed as broadly as possible on the slice with the lesion’s maximum diameter. SI of the spinal cord at the same level as the lesion was measured using 10-mm-diameter circle ROIs to calculate the tumor-to-spinal cord SI ratio (SIR). Apparent diffusion coefficient (ADC) values (× 10^−3^ mm^2^/s) were obtained from ADC maps by placing ROIs on solid tumor components.

Qualitative CT findings included hypodense, hyperintense, and calcified areas on unenhanced CT images, and heterogeneity on unenhanced and contrast-enhanced CT images. CT attenuations of hypodense, hyperdense, and calcified areas were defined as < 10HU, 50–200 HU, and > 200 HU, respectively.

Quantitative CT measurements included CT attenuation of solid components on unenhanced and contrast-enhanced CT images. A ROI within the tumor on CT images was placed to include as many solid components as possible while avoiding focal hypodense or unenhanced areas indicating necrosis or cystic change, focal hyperdense areas, and calcification.

### Statistical analysis

All statistical analyses were performed using the Statistical Package for the Social Sciences ver. 24.0 (IBM Corp., Armonk, NY, USA) or EZR (Saitama Medical Center, Jichi Medical University, Saitama, Japan). Fisher’s exact test was applied to compare differences in qualitative parameters (gender, location, configuration, heterogeneity, SI, focal unenhanced area, and time-intensity curve type) between the two groups. Interobserver variability in qualitative assessments was evaluated using Kappa statistics, where a Kappa value of ≤ 0.20 was interpreted as slight agreement, 0.21–0.40 as fair agreement, 0.41–0.60 as moderate agreement, 0.61–0.80 as substantial agreement, and ≥ 0.81 as almost perfect agreement. The Mann–Whitney *U* test was used for comparisons of quantitative variables (age, maximum lesion diameter, SIR, ADC value, and CT attenuation) between the two groups. Interobserver variability in quantitative parameters were assessed using the intraclass correlation coefficient (ICC), where ICC of ≤ 0.5 was interpreted as poor agreement, 0.50–0.75 as moderate agreement, 0.75–0.90 as good agreement, and ≥ 0.90 as excellent agreement. *p* values < 0.05 were considered significant.

## Results

### Patient characteristics

A total of 11 patients with head and neck myoepithelioma (age range, 22–88 years; mean age, 50.7 years; 5 men and 6 women) and 103 patients with parotid pleomorphic adenoma (age range, 19–87 years; mean age, 55.1 years; 38 men and 65 women) were included. Age (*p* = 0.337) and gender (*p* = 0.402) distributions were not significantly different between the two groups.

### MRI findings

**Fig. 1:**
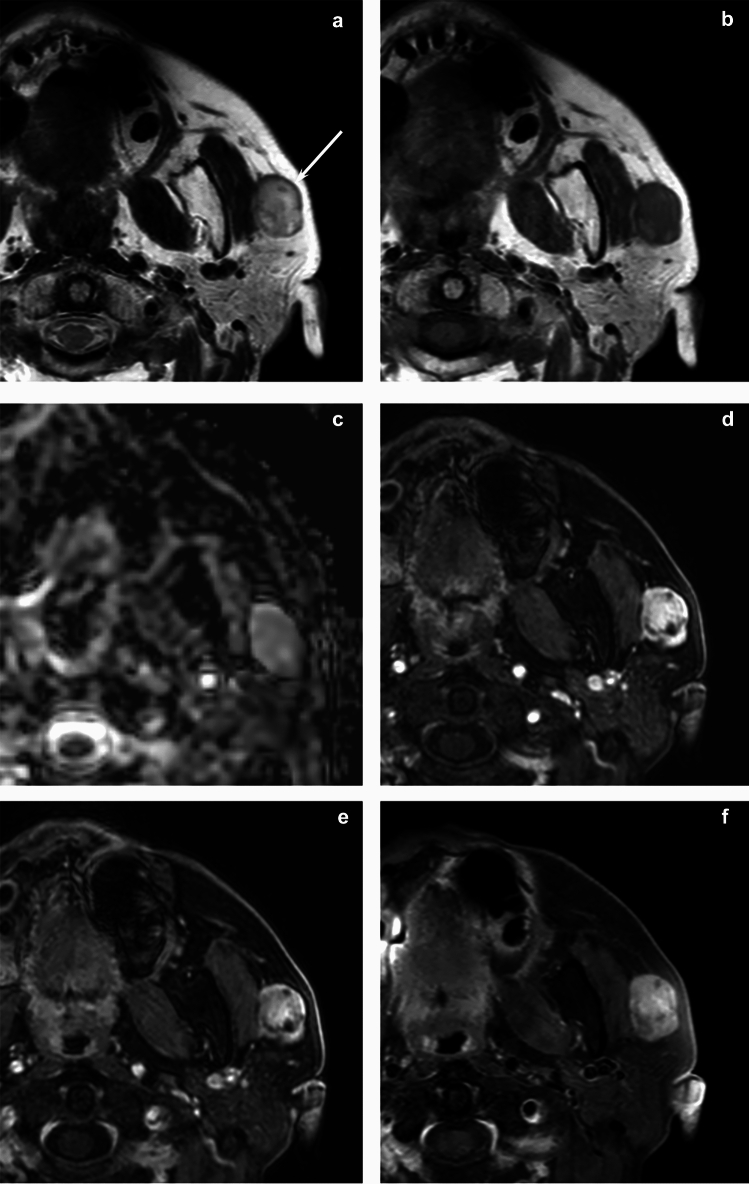
A 60-year-old woman with myoepithelioma of the left parotid gland. **a** T2-weighted image shows a heterogeneously mild hyperintense lesion (arrow). **b** T1-weighted image shows heterogeneously hypointense relative to the spinal cord. **c** ADC map shows high ADC value (1.68 × 10^−3^ mm^2^/s). **d** Fat-suppressed dynamic contrast-enhanced T1-weighted image 30 s after intravenous administration shows heterogeneously strong enhancement. **e.** Fat-suppressed dynamic contrast-enhanced T1-weighted image 180 s after intravenous administration shows mild washout. **f** Fat-suppressed contrast-enhanced T1-weighted image shows moderate enhancement with focal unenhanced areas

**Fig. 2:**
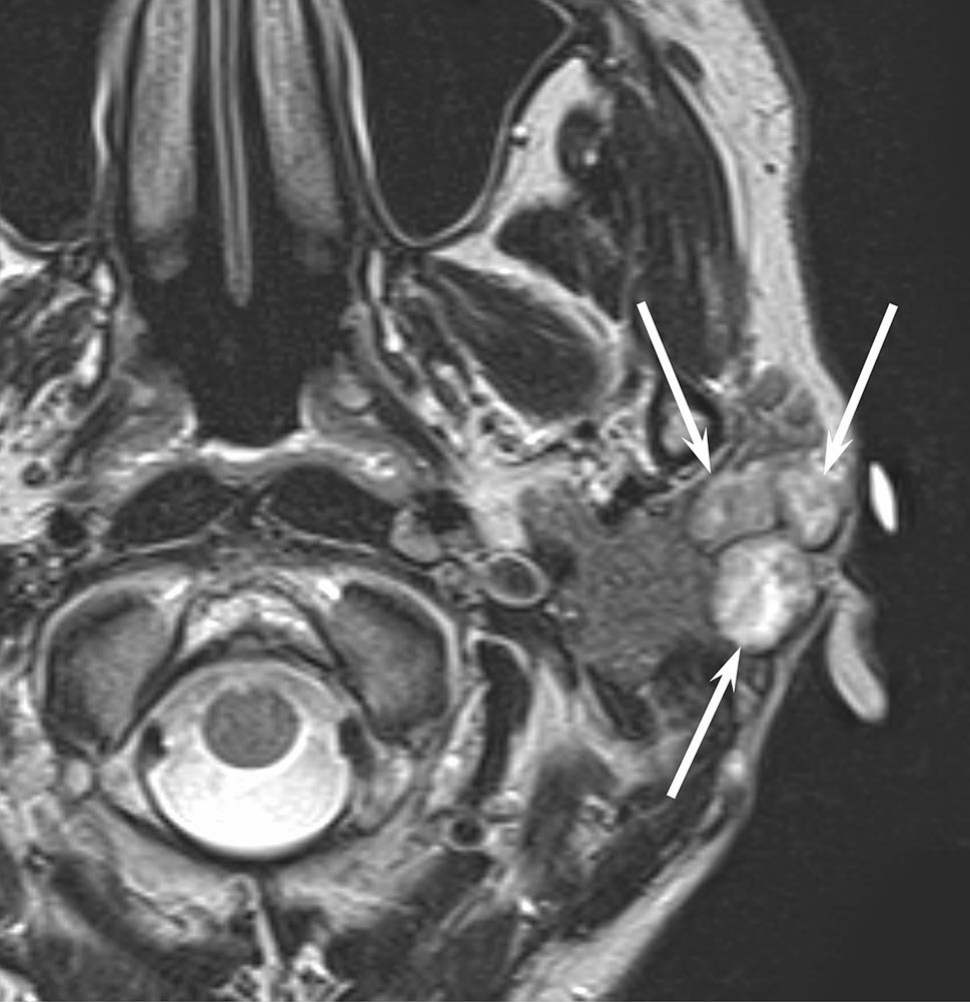
A 34-year-old woman with myoepithelioma of the left parotid gland. T2-weighted image shows a multinodular lesion with lobulated margin and intratumoral septa (arrows)

**Fig. 3:**
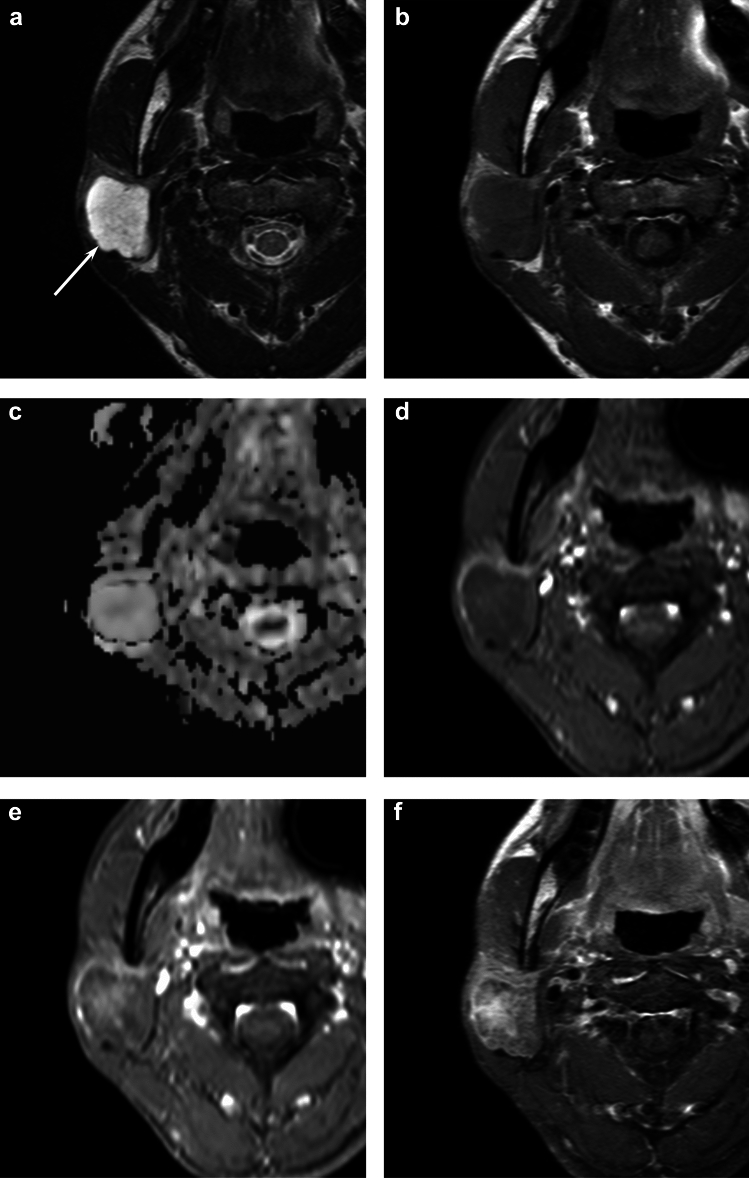
A 34-year-old woman with pleomorphic adenoma of the right parotid gland. **a** T2-weighted image shows a heterogeneously marked hyperintense lesion with lobulated margin (arrow). **b** T1-weighted image shows heterogeneously hypointense relative to the spinal cord. **c** ADC map shows extremely high ADC value (2.41 × 10^−3^ mm^2^/s). **d** Fat-suppressed dynamic contrast-enhanced T1-weighted image 30 s after intravenous administration shows minimal enhancement. **e** Fat-suppressed dynamic contrast-enhanced T1-weighted image 180 s after intravenous administration shows gradual enhancement. **f** Fat-suppressed contrast-enhanced T1-weighted image shows mild enhancement

**Fig. 4:**
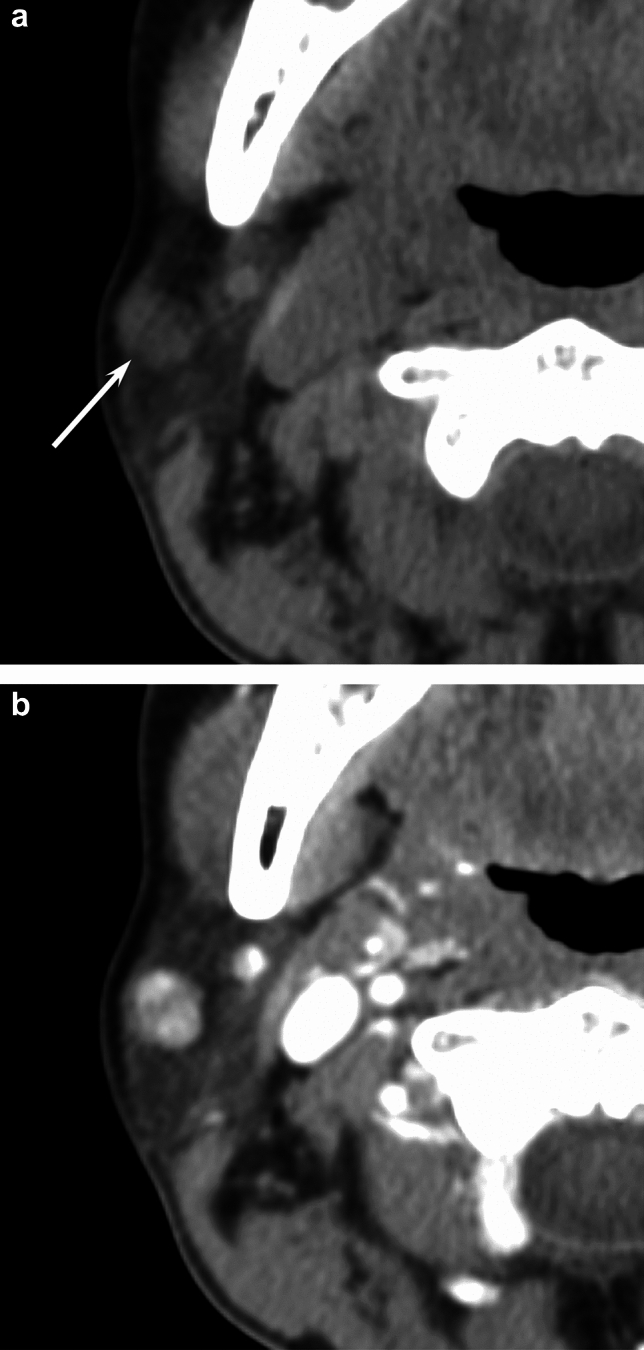
A 52-year-old woman with myoepithelioma of the right parotid gland. **a** Unenhanced CT image shows a hypodense lesion (arrow) (12 HU). **b** Contrast-enhanced CT image shows strong enhancement (104 HU)

**Fig. 5:**
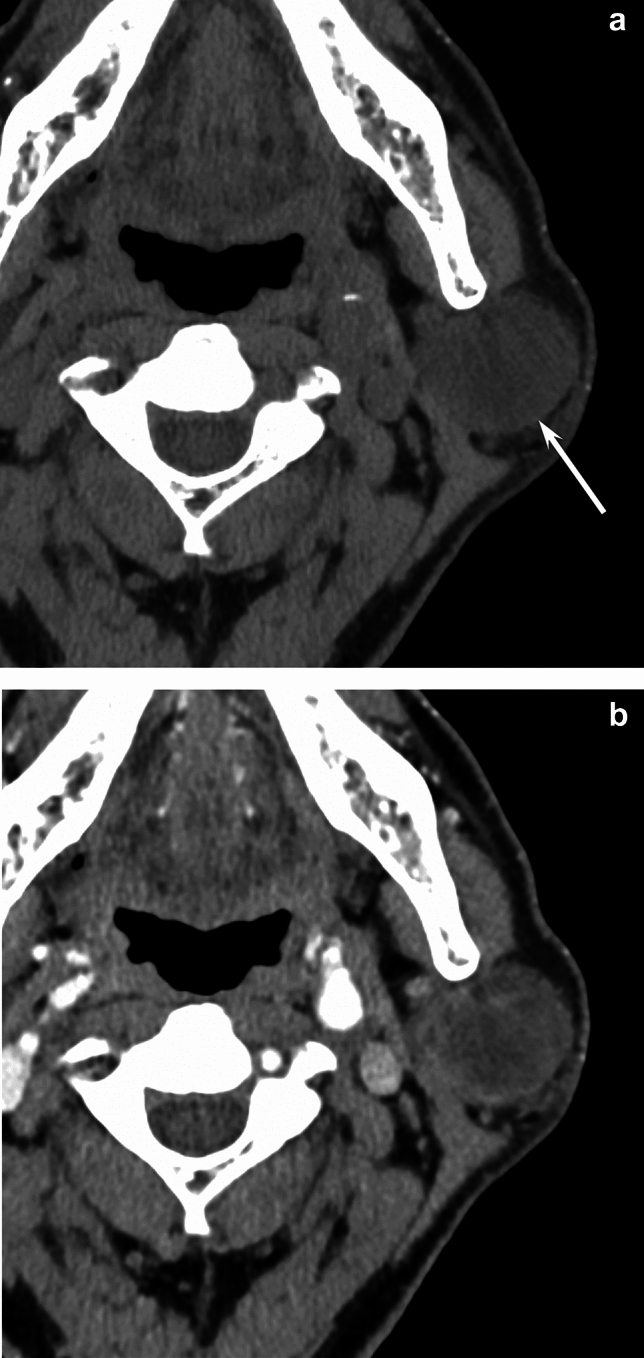
A 72-year-old man with pleomorphic adenoma of the left parotid gland. **a** Unenhanced CT image shows a hypodense lesion (arrow) (22 HU). **b** Contrast-enhanced CT image shows mild enhancement (38 HU)

A summary of MRI findings is presented in Table 1. Multinodular configuration (27% vs. 4%, *p* = 0.019), mild hyperintensity relative to the spinal cord on T2-weighted images (91% vs. 48%, *p* = 0.031), and focal unenhanced areas on fat-suppressed contrast-enhanced T1-weighted images (100% vs. 47%, *p* = 0.035) were significantly more frequent in myoepithelioma than in pleomorphic adenoma (Figs. 1, 2, 3, 4, and 5).

**Table 1:** MRI findings of myoepithelioma and pleomorphic adenoma

Parameter	Myoepithelioma (*n* = 11)	Pleomorphic adenoma (*n* = 103)	*p* value
Qualitative imaging findings			
Location: superficial	5 (83) (*n* = 6)	77 (75)	0.537
Location: superior	5 (83) (*n* = 6)	56 (54)	0.168
Lobulated margin	4 (36)	64 (62)	0.092
Multinodular configuration	3 (27)	4 (4)	0.019*
Capsule formation			0.053
Entire	8 (73)	49 (48)	
Partial	2 (18)	52 (50)	
Absence	1 (9)	2 (2)	
Intratumoral septa	4 (36)	21 (20)	0.197
T1WI			
SI of the lesion on T1WI			0.147
Hyperintensity	0 (0)	3 (3)	
Isointensity	8 (73)	43 (42)	
Hypointensity	3 (27)	57 (55)	
Heterogenous on T1WI	2 (18)	39 (38)	0.169
Hyperintense area on T1WI	2 (18)	9 (9)	0.287
T2WI			
SI of the lesion on T2WI			0.031*
Marked hyperintensity	1 (9)	47 (46)	
Mild hyperintensity	10 (91)	49 (48)	
Hypo- to isointensity	0 (0)	7 (7)	
Heterogenous on T2WI	9 (82)	92 (89)	0.365
Hypointense area on T1WI	2 (18)	21 (20)	0.611
CE-T1WI	(*n* = 5)	(*n* = 36)	
Enhancement of the lesion on FS CE-T1WI			0.409
Strong	0 (0)	8 (22)	
Moderate	5 (100)	21 (58)	
Mild	0 (0)	7 (19)	
Heterogenous on FS CE-T1WI	4 (80)	34 (94)	0.330
Focal unenhanced areas	5 (100)	17 (47)	0.035*
Time intensity curve pattern	(*n* = 3)	(*n* = 18)	0.080
Type A (peak time ≤ 120 s and WR ≥ 30%)	0 (0)	0 (0)	
Type B (peak time ≤ 120 s and WR < 30%)	2 (67)	2 (11)	
Type C (peak time > 120 s)	1 (33)	16 (89)	
Quantitative imaging findings			
Maximum lesion diameter (mm)	23.7 ± 8.8 (11–40)	23.9 ± 15.9 (9–156)	0.669
SIR of the lesion			
T1WI	0.95 ± 0.11	0.91 ± 0.13	0.311
T2WI	1.40 ± 0.39	1.68 ± 0.47	0.034*
FS CE-T1WI	3.18 ± 2.10	2.17 ± 0.46	0.202
ADC value (× 10^−3^ mm^2^/s)	1.38 ± 0.23	1.68 ± 0.36	0.047*

Qualitative data are numbers of patients with percentages in parentheses

Quantitative data are expressed as mean ± standard deviation with ranges in parentheses

*T1WI* T1-weighted images, *SI* signal intensity, *T2WI* T2-weighted images, *FS CE-T1WI* fat-suppressed contrast-enhanced T1-weighted images, *WR* washout ratio, *SIR* signal intensity ratio, *ADC* apparent diffusion coefficient

^*^Significant differences in the values were observed between myoepithelioma and pleomorphic adenoma (*p* < 0.05)

In contrast, marked hyperintensity relative to the spinal cord on T2-weighted images (46% vs. 9%, *p* = 0.031), higher SIRs on T2-weighted images (1.68 ± 0.47 vs. 1.40 ± 0.39, *p* = 0.034), and higher apparent diffusion coefficient (ADC) values (1.68 ± 0.36 vs. 1.38 ± 0.23 × 10^−3^ mm^2^/s, *p* = 0.047) were significantly more common in pleomorphic adenoma than in myoepithelioma.

An entire capsule formation (73% vs. 48%, *p* = 0.053) and type B time-intensity curve (time of peak enhancement ≤ 120 s, washout ratio < 30%) (67% vs. 11%, *p* = 0.080) were more frequent in myoepithelioma than in pleomorphic adenoma, but differences were marginal. No other MRI differences reached significance.

### CT findings

A summary of CT findings is presented in Table 2. Contrast-enhanced CT attenuation was significantly higher in myoepithelioma than in pleomorphic adenoma (93.3 ± 10.5 vs. 59.2 ± 22.8 HU, *p* = 0.028) (Figs. 3 and 5). No other CT differences reached significance.

**Table 2:** CT findings of myoepithelioma and pleomorphic adenoma

Parameter	Myoepithelioma (*n* = 7)	Pleomorphic adenoma (*n* = 29)	*p* value
Qualitative imaging findings			
Unenhanced CT			
Hypodense areas	2 (29)	10 (34)	0.571
Hyperdense areas	2 (29)	2 (7)	0.163
Calcified areas	0 (0)	3 (10)	0.512
Heterogenous	4 (57)	16 (55)	0.631
Contrast-enhanced CT	(*n* = 3)	(*n* = 10)	
Heterogenous	1 (33)	3 (30)	0.706
Quantitative imaging findings			
Unenhanced CT attenuation (HU)	33.6 ± 13.4	31.3 ± 9.6	0.562
Contrast-enhanced CT attenuation (HU)	93.3 ± 10.5	59.2 ± 22.8	0.028*

Qualitative data are numbers of patients with percentages in parentheses

Quantitative data are expressed as mean ± standard deviation

*HU* Hounsfield Unit

^*^Significant differences in the values were observed between myoepithelioma and pleomorphic adenoma (*p* < 0.05)

### Interobserver variability

The κ values ranged from 0.41 to 1.00, indicating moderate to almost perfect agreement between the two radiologists. The ICCs ranged from 0.76 to 0.99, indicating good-to-excellent agreement between the two reviewers.

## Discussion

Our results indicate that multinodular configuration, lower SIR on T2-weighted images, lower ADC values, focal unenhanced areas, and higher contrast-enhanced CT attenuation were more common in myoepithelioma than in pleomorphic adenoma.

Histologically, myoepitheliomas consist almost exclusively of sheets, islands, or cords of myoepithelial cells with spindle, plasmacytoid, or clear cytoplasmic features, and lack epithelial components. They arise from neoplastic myoepithelial or basket cells and are made up of numerous cellular elements including smooth muscle actin, myosin, and intermediate filaments [14]. Their dense cellularity and fibrous stroma explain the lower T2 SI and lower ADC values found in the present study. Previous reports similarly note low ADC values (1.12 and 0.76 × 10^−3^ mm^2^/s) [10]. In contrast, typical pleomorphic adenomas show marked hyperintensity on T2-weighted images due to abundant myxochondroid stroma [15]. Therefore, it is not difficult to differentiate between myoepithelioma and typical pleomorphic adenoma.

**Figs. 6 and 7:**
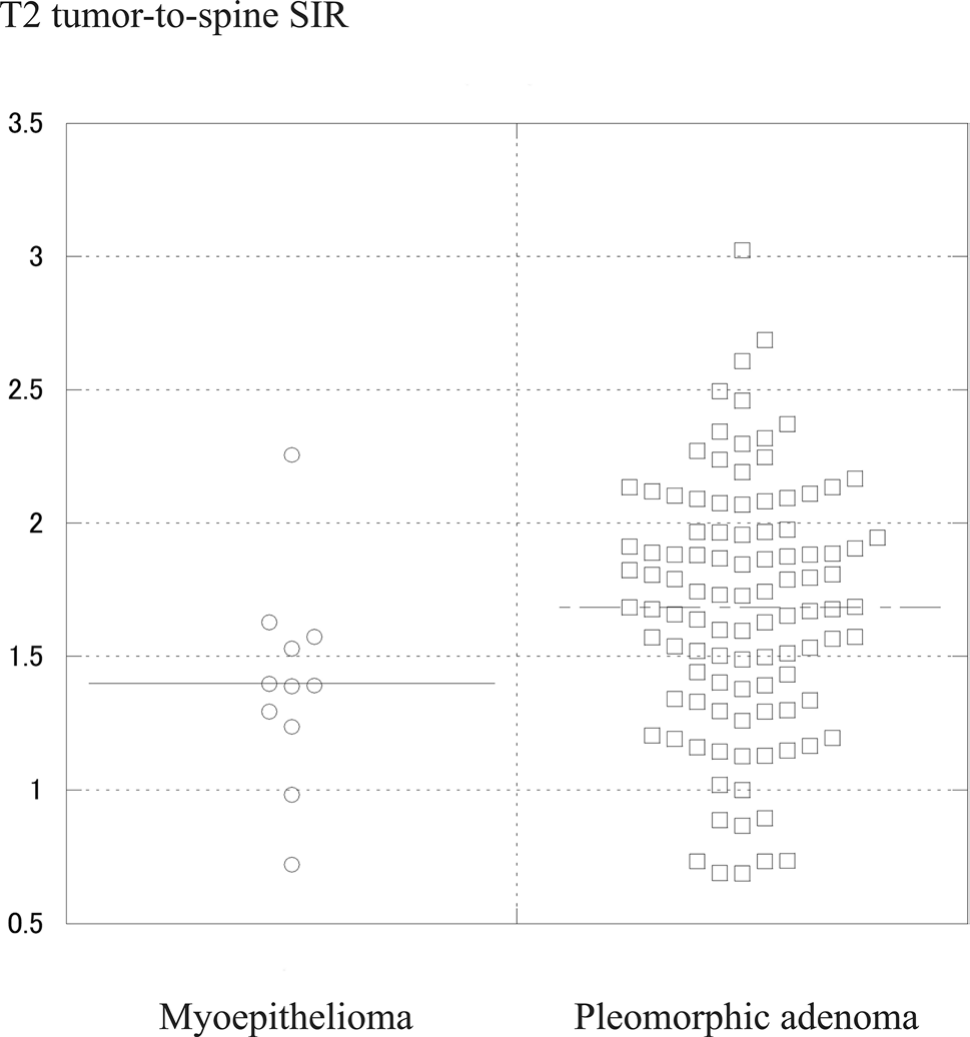

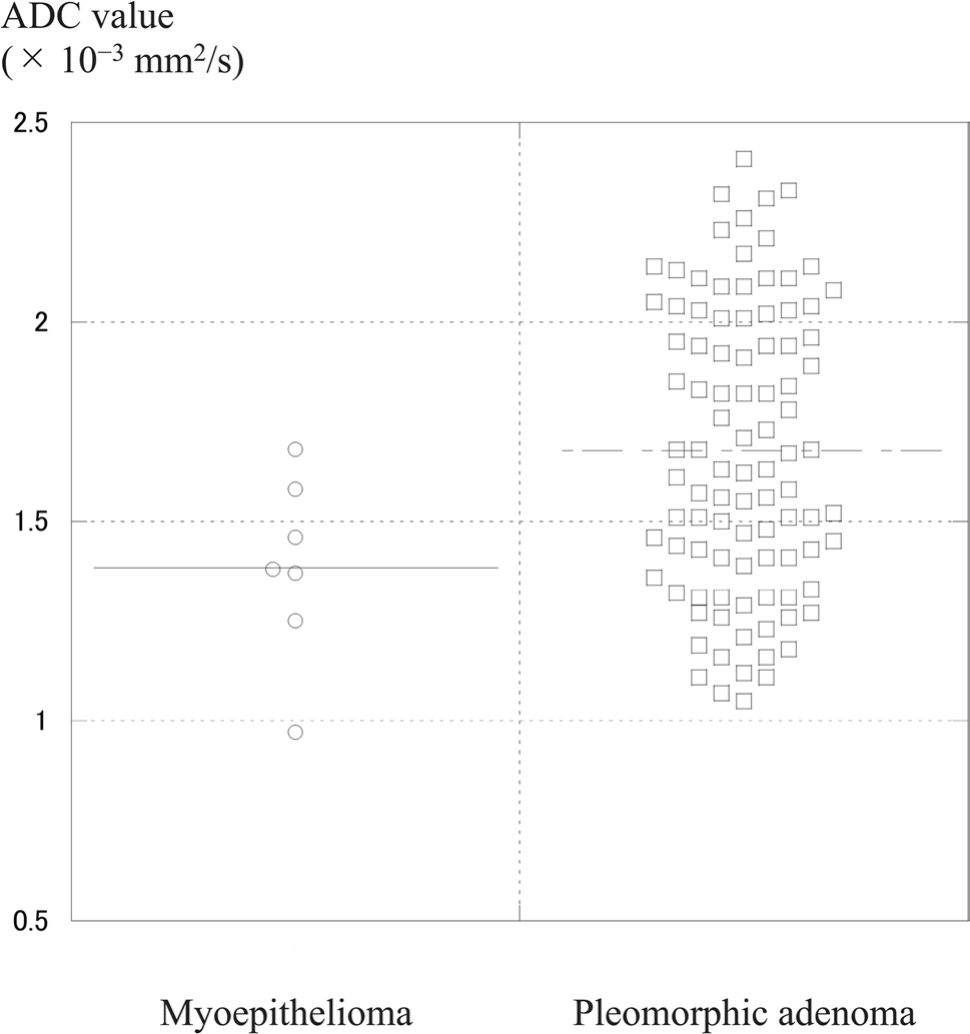
Beeswarm plots of SIRs on T2-weighted images (Fig. 6) and ADC values (Fig. 7). The considerable overlap between myoepithelioma and pleomorphic adenoma in intermediate SIRs on T2-weighted images and intermediate ADC values, while tumors with high SIRs on T2-weighted images and high ADC values are more likely pleomorphic adenomas

Cellular pleomorphic adenomas, comprising 36.5% of all cases, have minimal stroma and predominantly epithelial cells, leading to intermediate SI on T2-weighted images and intermediate ADC values [15]. Therefore, MRI findings of myoepithelioma may overlap with those of cellular pleomorphic adenomas. Indeed, the beeswarm plots revealed the considerable overlap between myoepithelioma and pleomorphic adenoma in intermediate SIRs on T2-weighted images and intermediate ADC values (Figs. 6 and 7). However, tumors with high SIRs on T2-weighted images and high ADC values are more likely pleomorphic adenomas (Figs. 6 and 7).

In the present study, the observed higher contrast-enhanced CT attenuation in myoepithelioma aligns with prior reports where the average unenhanced CT attenuation ranged from 30 to 44HU, while post-contrast CT attenuation (35–40 s after intravenous administration of contrast agent) ranged from 90 to 141HU [12, 13, 16]. In addition, mean post-contrast CT attenuation on delayed phase (180 s after the onset of contrast injection) was 102HU [16]. Factors influencing enhancement include stroma composition, vascularity, and histological cell type [11]. Cellular myoepitheliomas with fibrous stroma enhance more strongly than those with myxoid stroma [11]. Conversely, 69.4% of cases with pleomorphic adenoma showed weak enhancement (< 70 HU) (30 s after intravenous administration of contrast agent) [17]. The weak enhancement in the early phase and delayed enhancement with a prolonged time of peak enhancement are usually caused by low vascularity and abundant myxochondroid stroma within pleomorphic adenoma [15]. Therefore, contrast-enhanced CT attenuation in the early phase would contribute to the accurate differentiation between myoepithelioma and pleomorphic adenoma, reflecting differences in vascularity. In addition, hypervascular tumors tend to be heterogeneous and have focal unenhanced areas.

Although multinodular configuration is rare in salivary gland tumors, it was observed in 27% of myoepitheliomas in this study. Multinodular structure with internal septa was also observed in 57% of epithelial–myoepithelial carcinomas [18]. This feature reflects the multinodular growth pattern shared by myoepithelial tumors, including myoepithelioma and epithelial-myoepithelial carcinoma [19]. In contrast, pleomorphic adenomas more often show lobulated margins without multinodular configuration, suggesting that multinodular configuration would be characteristic of myoepithelial tumors.

This study has several limitations. First, the small sample size is serious limitation of the present study. In particular, myoepithelioma is a rare tumor, and it was difficult to collect sufficient cases; therefore, we used cases currently available. To increase the number of cases of myoepithelioma, the locations of myoepithelioma in the present study included various head and neck regions. Second, when comparing the number of cases collected during the same period to show the difference in frequency between myoepithelioma and pleomorphic adenoma, a considerable imbalance of the number of cases between the groups occurred. The imbalance between the groups may undermine the stability and generalizability of the conclusions. Therefore, future verification studies are required, such as increasing the number of myoepithelioma and conducting multicenter studies. Third, we believe that significant results based on marginal *p* values and small differences should be interpreted with caution in terms of their practical significance, as type I errors (false positives) due to chance are likely to occur when the sample size is insufficient. However, as sample sizes increase, even small differences may become statistically significant. Fourth, narrow comparison scope is also limitation of the present study. Further investigation is required, including other differential diagnoses such as Warthin tumor or oncocytoma, which often pose a greater diagnostic challenge on imaging. Fifth, the MRI findings were obtained using MRI scanners with different magnetic field strengths (1.5 T or 3 T), which could potentially influence the calculated ADC values. Finally, not all patients underwent complete MRI or CT protocols due to the retrospective nature of this study. Despite this, the present study could reveal the characteristic MRI and CT features of myoepithelioma.

In conclusion, differentiating myoepithelioma from pleomorphic adenoma remains challenging due to overlapping features. However, multinodular configuration, lower SIR on T2-weighted images, focal unenhanced areas, lower ADC values, and higher early phase contrast-enhanced CT attenuation may aid in distinguishing myoepithelioma, reflecting its hypercellular and hypervascular nature.
